# Morphological and Dynamic Contrast-Enhanced MRI Characteristics of Breast Imaging Reporting and Data System (BI-RADS) 3 and Above Breast Masses With Color Doppler and Histopathological Correlation

**DOI:** 10.7759/cureus.101957

**Published:** 2026-01-21

**Authors:** Shivya Parashar, Sangeeta Saxena, Jitendra Sharma, Sanjeev Shukla

**Affiliations:** 1 Radiodiagnosis, All India Institute of Medical Sciences Bhopal, Bhopal, IND; 2 Radiodiagnosis, Government Medical College (GMC) Kota, Kota, IND; 3 Radiology, All India Institute of Medical Sciences Bhopal, Bhopal, IND; 4 Radiology, Himalayan Diagnostics, Bhopal, IND

**Keywords:** birads 3 and above masses, breast ultrasound, color doppler, diffusion imaging, histopathology, mr mammography

## Abstract

This prospective study evaluated the morphological characteristics, enhancement patterns, and diffusion-weighted imaging (DWI) features of Breast Imaging Reporting and Data System (BI-RADS) 3 and above breast masses on MRI, correlated the imaging findings with color Doppler, and assessed diagnostic performance. Thirty-eight women underwent ultrasound, color Doppler, and breast MRI with DWI, with imaging findings compared against histopathology as the reference standard. MRI demonstrated high sensitivity for malignant features, including irregular margins, heterogeneous or rim enhancement, and washout kinetic patterns. The addition of DWI significantly improved specificity. Color Doppler showed limited diagnostic utility, with meaningful diagnostic value primarily observed when the resistive index exceeded 0.7. The combined evaluation of MRI morphology, enhancement kinetics, and DWI provides high diagnostic accuracy in possibly malignant breast lesions.

## Introduction

The aim of this study was to evaluate the morphological characteristics and enhancement patterns of suspicious breast masses on magnetic resonance imaging (MRI). The study also sought to correlate MRI findings with colour Doppler and histopathological results, to assess the role of diffusion-weighted imaging (DWI) in improving the specificity of breast MRI, and to analyze the MRI characteristics of Breast Imaging Reporting and Data System (BI-RADS) 3 and above breast masses.

Breast cancer has emerged as the most common cancer among women in India, surpassing cervical cancer in both incidence and mortality, as reported by national and international cancer registries. Data from GLOBOCAN and Indian cancer registries demonstrate a growing epidemiological shift, with breast cancer accounting for a higher proportion of female malignancies over the past decade, reflecting an increasing public health burden [1].

Early and accurate diagnosis plays a critical role in reducing breast cancer-related mortality, as treatment outcomes are significantly improved when the disease is detected at an early stage. Despite advances in diagnostic imaging, late presentation remains a major challenge in India due to limited awareness, sociocultural barriers, stigma, and low participation in screening programs, particularly in rural and underserved populations. Epidemiological studies indicate that a substantial proportion of patients present with advanced-stage disease (Stage III or IV) at the time of initial diagnosis, largely attributable to diagnostic delays and poor health-seeking behaviour [2].

Mammography, ultrasound, and MRI play complementary roles in the evaluation of suspicious breast lesions. Mammography remains the only imaging modality proven in large randomized controlled trials to reduce breast cancer mortality and continues to serve as the cornerstone of population-based screening [3]. However, its diagnostic accuracy is reduced in women with dense breast tissue, invasive lobular carcinoma, and postoperative breasts, and in individuals with genetic predispositions, such as BRCA mutations, necessitating the use of adjunct imaging techniques [3,4]. Breast MRI offers the highest sensitivity for breast cancer detection among currently available imaging modalities and has become an integral component of modern breast imaging practice [4]. Radiologic-pathologic correlation remains essential to ensure diagnostic accuracy, as histopathology continues to serve as the definitive reference standard for the diagnosis of breast masses [5].

## Materials and methods

Study design

This was a prospective observational study conducted to evaluate the morphological characteristics, enhancement patterns, and DWI features of suspicious breast masses on MRI, with correlation to color Doppler findings and histopathological results.

Study population and sample size

The study included 38 women aged 19 to 75 years who presented with probably malignant breast lesions on initial imaging evaluation. All participants underwent ultrasound examination with color Doppler followed by dynamic contrast-enhanced breast MRI with DWI. Patients with BI-RADS category 1, 2, or 6 lesions, those who had received chemotherapy, those with a history of recent breast biopsy, and pregnant women were excluded from the study. Histopathological examination served as the reference standard for final diagnosis in all cases.

Ultrasound and color Doppler evaluation

Gray-scale ultrasound and color Doppler evaluation were performed prospectively for all 38 breast masses. Color Doppler assessment was performed for all breast masses to evaluate both quantitative and qualitative vascular characteristics. Quantitative parameters included measurement of the resistive index, with values greater than 0.7 considered suggestive of malignancy, and peak systolic velocity, with a threshold of more than 20 cm/s. Qualitative assessment focused on the pattern and distribution of vascularity, including the presence of centrally located vessels with branching and penetrating morphology, higher velocity flow manifested by color aliasing, the proportion of the lesion demonstrating color mapping, and the angle of vessel entry into the mass, with a perpendicular course considered a suspicious feature. These parameters were systematically analyzed and correlated with histopathological findings.

MRI protocol

Breast MRI examinations were performed using a dedicated 16-channel breast coil with patients positioned prone. The imaging protocol included T2-weighted sagittal and axial fat-suppressed sequences and T1-weighted axial non-fat-suppressed sequences. A sagittal fat-suppressed T2-weighted fast spin-echo sequence was acquired with a repetition time/echo time of 4500/85 ms, a field of view of 34 cm, a matrix size of 256 × 192, and a slice thickness of 5 mm with a 1 mm interslice gap.

Dynamic contrast-enhanced imaging was performed using a three-dimensional, axial, fat-suppressed, T1-weighted, fast gradient-recalled-echo (GRE) sequence (dynamic e-thrive) with a slice thickness of 2 mm and no interslice gap. Images were obtained before and during five post-contrast phases following a bolus intravenous injection of 0.1 mmol/kg of gadopentetate dimeglumine. Each dynamic phase consisted of 74 images acquired over 90 seconds. Imaging parameters included minimum echo time, preparation time of 40 ms, a flip angle of 10 degrees, and a field of view of 34 cm. Subtraction images, maximum intensity projection images, and computer-aided detection analysis were generated for lesion evaluation.

DWI and apparent diffusion coefficient (ADC) analysis

DWI was performed as part of the MRI protocol, and ADC maps were generated using b values of 0 and 1000 s/mm². ADC values were measured for each lesion and analysed to assess their contribution to improving the specificity of breast MRI in differentiating benign from malignant lesions. For diffusion-weighted imaging analysis, an ADC cut-off value of < 1.25 × 10⁻³ mm²/s was used to differentiate malignant from benign breast lesions. This threshold was selected based on prior published literature and was applied uniformly across the study cohort. The diagnostic performance of this cut-off was subsequently evaluated through statistical analysis with histopathology serving as the reference standard.

Image interpretation

All imaging findings were interpreted using the American College of Radiology (ACR) BI-RADS MRI lexicon. Lesions were assessed for morphological features, enhancement characteristics, and diffusion properties, and categorized according to BI-RADS criteria for both mass and non-mass enhancement.

Histopathological correlation

Histopathological examination of tissue samples obtained through biopsy or surgical excision served as the definitive reference standard. Imaging findings were correlated with histopathological diagnoses for final analysis.

Statistical analysis

Statistical analysis was performed to evaluate the diagnostic performance of MRI, DWI, and color Doppler parameters. Sensitivity, specificity, positive predictive value, negative predictive value, and diagnostic accuracy were calculated. Comparative analysis was performed using appropriate statistical tests, including the McNemar test for comparison of paired proportions, with a p-value of less than 0.05 considered statistically significant.

Ethics statement

The study was conducted in accordance with the ethical standards of the institutional research committee. Written informed consent was obtained from all participants prior to inclusion in the study.

## Results

Patient characteristics

A total of 38 patients were included in the study, all of whom underwent ultrasonography (USG) with color Doppler, breast MRI, and histopathological examination. Histopathology revealed 25 malignant lesions and 13 benign lesions. Among benign lesions, fibroadenoma was the most common, whereas invasive ductal carcinoma (IDC) predominated among malignant lesions.

Color Doppler ultrasound findings

Of the 25 malignant lesions, 9 (36%) were avascular, while 6 of 13 (46%) benign lesions showed no detectable vascularity. Using vascularity as a criterion for malignancy yielded a sensitivity of 64% and a specificity of 46% (Table 1).

**Table 1:** Distribution of the lesions according to the presence of vascularity

	Benign	Malignant
Avascular	6	9
Vascular	7	16
Total	13	25

**Figure 1:**
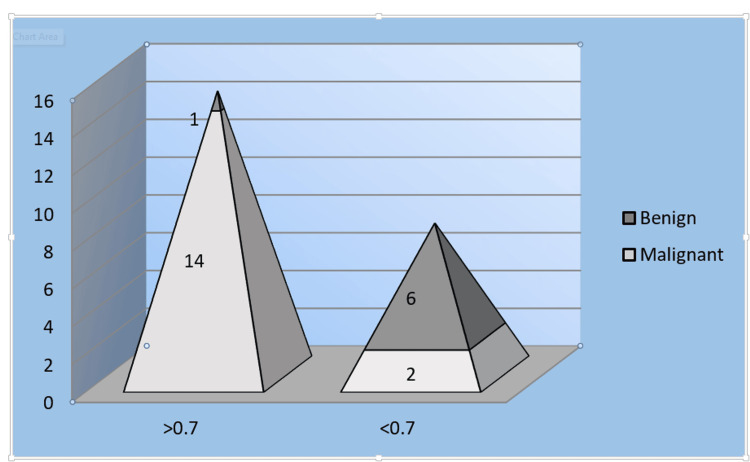
Graphic representation of the distribution of the RI value in lesions RI: resistive index

The resistive index (RI) was evaluated in vascular lesions. An RI of >0.7 was observed in 14 of 16 malignant lesions and in only 1 benign lesion, whereas an RI of <0.7 was noted in 6 benign lesions and 2 malignant lesions. Only one malignant vascular lesion had an RI of <0.7, suggesting that RI is a highly specific marker of malignancy (Figure 1).

In the present study, color Doppler evaluation focused on both the presence and pattern of vascularity within breast lesions. Vascularity was qualitatively categorized as peripheral, central, or combined (central with peripheral flow). The angle of vessel entry was also assessed, as penetrating vessels entering the lesion perpendicularly are more frequently associated with malignancy. Malignant lesions in our study more commonly demonstrated central or mixed vascularity with penetrating vessels, consistent with tumor-related angiogenesis, whereas benign lesions predominantly showed peripheral or absent flow.

Quantitative Doppler assessment was performed using spectral Doppler parameters, including RI, pulsatility index (PI), and peak systolic velocity (PSV), obtained from the most prominent intra- or perilesional vessel. These Doppler parameters were correlated with histopathological findings to evaluate their diagnostic performance. Sensitivity, specificity, positive predictive value, negative predictive value, and diagnostic accuracy were calculated, and statistically derived cut-off values were used to compare benign and malignant lesions and assess their discriminatory potential.

Peripheral vascularity was observed in eight malignant and four benign lesions, while combined central and peripheral vascularity was seen in two malignant and six benign lesions. Overall, color Doppler demonstrated a sensitivity of 56%, specificity of 53%, and a positive predictive value of 93.33% for detecting malignant lesions.

MRI findings

Among the 38 cases, 35 lesions were masses, and 3 showed non-mass-like enhancement. The most common lesion shape was irregular, with benign lesions predominantly oval and malignant lesions irregular.

**Figure 2:**
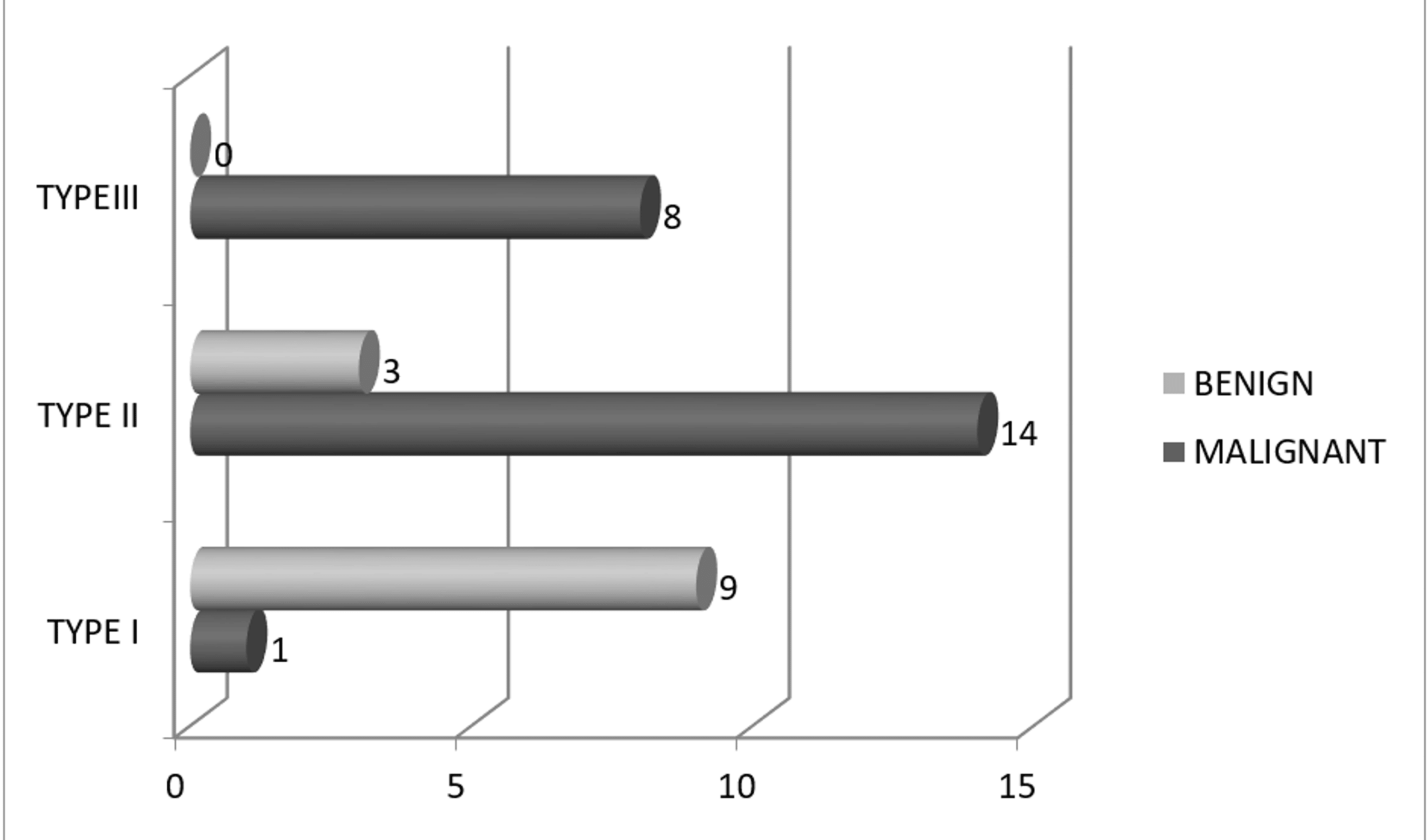
Distribution of mass lesions according to the type of initial enhancement curve in a lesion on dynamic contrast studies

Mass margins, according to the BI-RADS lexicon, were classified as circumscribed and not circumscribed (irregular, or spiculated). Circumscribed margins were present in 9 of 13 benign masses, and no benign lesion showed spiculated margins (Figure 2).

Initial enhancement patterns on dynamic contrast-enhanced MRI were classified as slow, medium, or rapid. In benign lesions, slow enhancement was most common (6 cases), whereas rapid enhancement predominated in malignant lesions (19 cases).

For statistical analysis and correlation with histopathology, enhancement characteristics were dichotomized to derive diagnostic performance indices. Initial enhancement was categorized as positive (suspicious/malignant) when medium or rapid enhancement was observed, while slow enhancement was considered negative (benign). Similarly, delayed enhancement patterns were grouped such that type II (plateau) and type III (washout) curves were analyzed as malignant, whereas type I (persistent) curves were considered benign, in accordance with established BI-RADS MRI conventions.

These dichotomized imaging variables were subsequently correlated with histopathological outcomes to calculate sensitivity, specificity, positive predictive value, negative predictive value, and overall diagnostic accuracy.

Delayed kinetic curves were categorized as Type I (progressive), Type II (plateau), or Type III (washout). Benign lesions most frequently demonstrated Type I curves, while malignant lesions primarily showed Type II (14 cases) and Type III (8 cases) curves.

Among 38 lesions, heterogeneous enhancement was predominantly associated with malignancy (13/16, 81.3%), while homogeneous enhancement was more frequently seen in benign lesions (4/6, 66.7%). Solid lesions with dark septations were exclusively benign (3/3). Cystic lesions with enhancing mural nodules (3/4, 75%) and rim enhancement (7/9, 77.8%) were more commonly malignant (Table 2).

**Table 2:** Distribution of malignant and benign lesions according to the pattern of internal enhancement in lesions on dynamic MRI scan

PATTERN	BENIGN	MALIGNANT	TOTAL
Homogeneous	4	2	6
Heterogeneous	3	13	16
Solid with dark septations	3	0	3
Cyst with an enhancing mural nodule	1	3	4
Rim enhancement	2	7	9
Total	13	25	38

The sensitivity of MR mammography was significantly higher than that of color Doppler for detecting malignant breast lesions (McNemar test, p = 0.00001).

When comparing multiparametric MRI (dynamic contrast-enhanced (DCE) + morphology + DWI) with DCE + morphology alone, the addition of DWI significantly improved sensitivity (McNemar test, p = 0.007).

## Discussion

The findings of the present study reaffirm the established diagnostic strengths of breast MRI, particularly in characterizing suspicious breast masses. Dynamic contrast-enhanced MRI offers superior sensitivity owing to its ability to assess both morphological and kinetic features. In our study, malignant lesions demonstrated typical high-risk descriptors, including irregular and spiculated margins, heterogeneous or rim enhancement patterns, and rapid wash-in followed by washout kinetics. These features correlate with increased angiogenesis and chaotic tumor vasculature, which have been extensively documented in prior research.

The significance of DWI in breast MRI has increased considerably in the last decade. Malignant lesions showed significantly lower ADC values, reflecting restricted diffusion due to high cellular density [6]. Multiple large-scale studies have shown ADC thresholds between 0.9-1.3 × 10⁻³ mm²/s to be effective in differentiating benign from malignant lesions [7,8]. Our findings agree with this range, supporting the utility of DWI as a non-contrast functional biomarker. The inclusion of DWI is particularly valuable where contrast administration is contraindicated, such as in renal impairment or pregnancy.

Color Doppler, although helpful as a complementary tool, demonstrated limited diagnostic performance in this study. While vascularity alone was not a reliable predictor, RI > 0.7 was strongly associated with malignancy. This aligns with previous observations that malignant lesions exhibit increased vascular resistance due to neovascular architectural distortions [9,10]. However, Doppler findings should not be used in isolation, as benign lesions such as fibroadenomas usually show internal vascularity.

**Figure 3:**
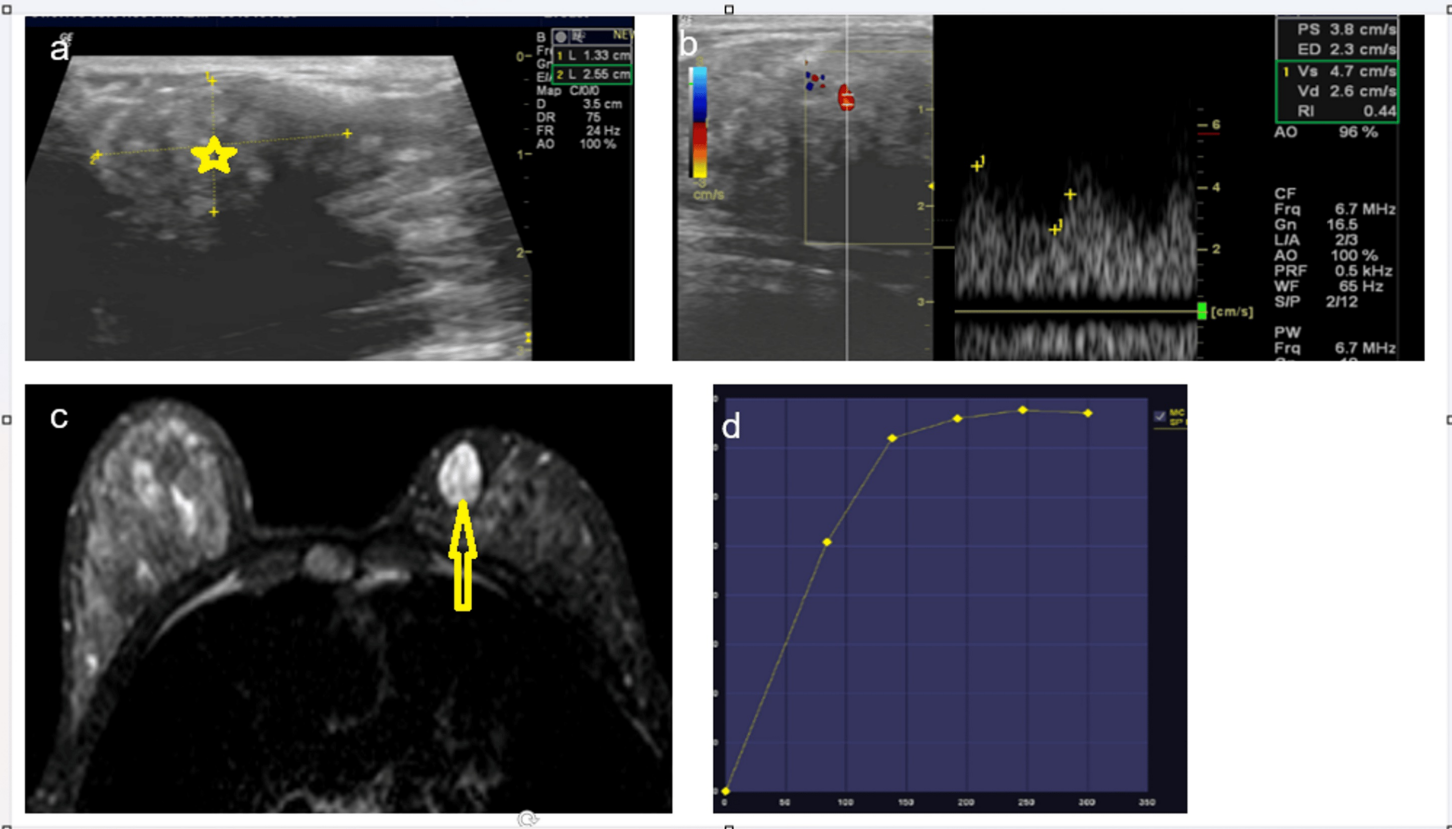
(A–D): Imaging features of fibroadenoma in a 20-year-old female involving the right breast (A) Gray-scale ultrasound shows a well-defined, oval, heterogeneous mass with circumscribed margins and parallel orientation (yellow star). (B) Color Doppler with spectral analysis demonstrates minimal internal vascularity with low-resistance flow (RI<0.7), favouring a benign etiology. (C) Axial post-contrast dynamic contrast-enhanced MRI reveals a well-circumscribed lesion with homogeneous enhancement and dark internal septations (yellow open arrow). (D) Time–intensity curve shows a Type I (persistent) enhancement pattern, consistent with benign pathology.

The multimodality imaging features of fibroadenoma observed in the present study, as illustrated in Figure 3, exemplify the typical benign imaging phenotype.

The lesion demonstrated benign morphology on ultrasound with minimal internal vascularity on color Doppler, correlating with the known low angiogenic potential of fibroadenomas. On dynamic contrast-enhanced MRI, homogeneous enhancement with a persistent (Type I) kinetic curve was observed, a pattern consistently associated with benign breast lesions. These combined imaging characteristics highlight the complementary role of ultrasound, Doppler, and contrast-enhanced MRI in improving diagnostic confidence and specificity in BI-RADS 4 lesions, thereby potentially reducing unnecessary biopsies, particularly in younger patients.

**Figure 4:**
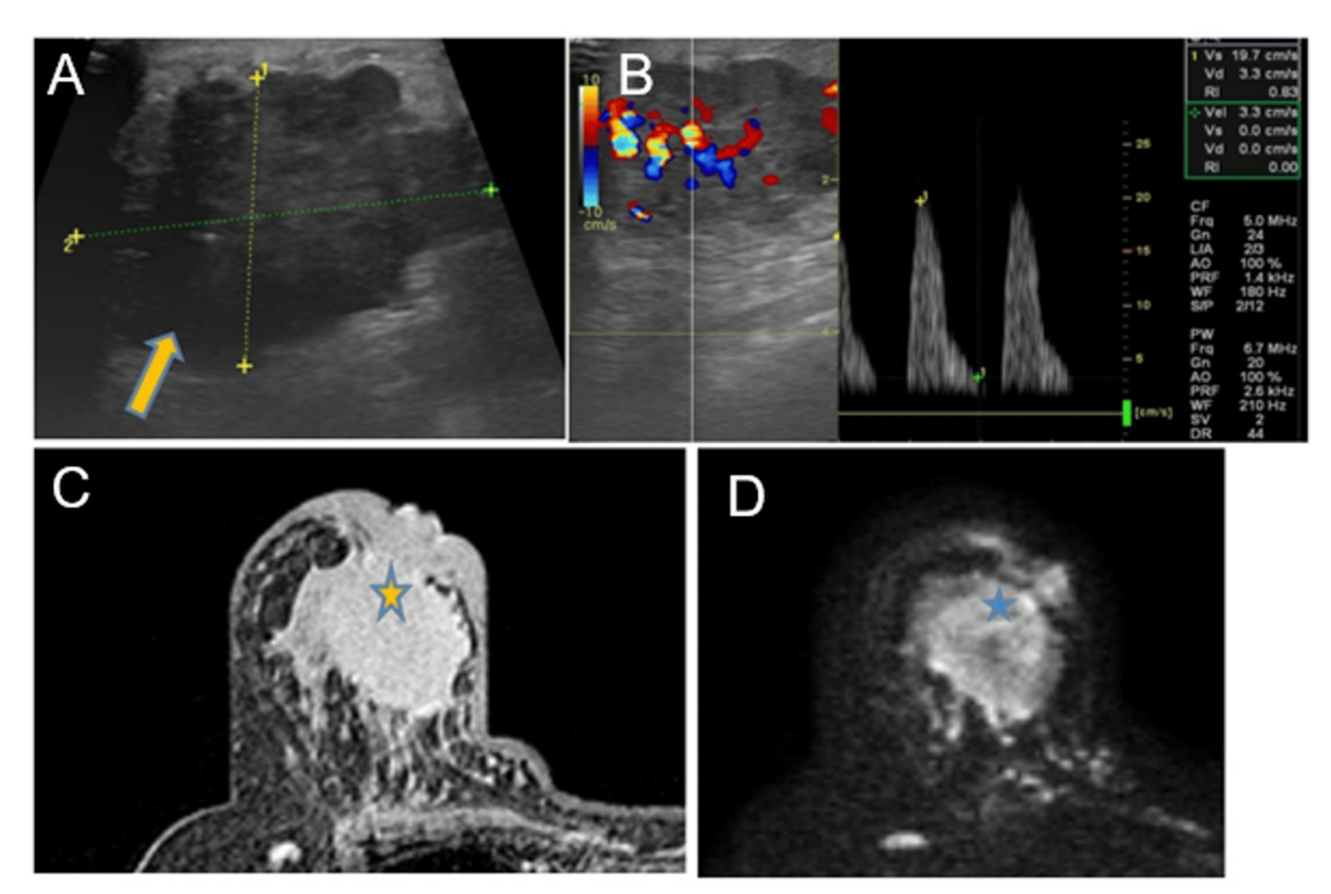
(A-D) Ultrasound, color Doppler, and MRI correlation in a malignant breast lesion (A) Gray-scale ultrasound shows an irregular, hypoechoic breast mass with microlobulated margins (yellow arrow). (B) Color Doppler demonstrates increased vascularity with high-resistance flow with an elevated resistive index (RI >0.7). (C) Contrast-enhanced MRI depicts an irregular heterogeneously enhancing mass causing infiltration of skin and nipple areola complex (yellow star). (D) Diffusion-weighted imaging shows diffusion restriction within the lesion (blue star), consistent with high cellularity.

Another illustrated case (Figure 4) demonstrates the value of multimodality imaging in characterizing malignant breast lesions. The presence of irregular margins on gray-scale ultrasound with increased ascularity and high-resistance Doppler flow reflects tumor-related neoangiogenesis. Corresponding MRI findings of heterogeneous enhancement and diffusion restriction further support malignancy by indicating disorganized tumor vascularity and high cellular density, underscoring the complementary role of ultrasound, Doppler, and MRI in improving diagnostic confidence.

**Figure 5:**
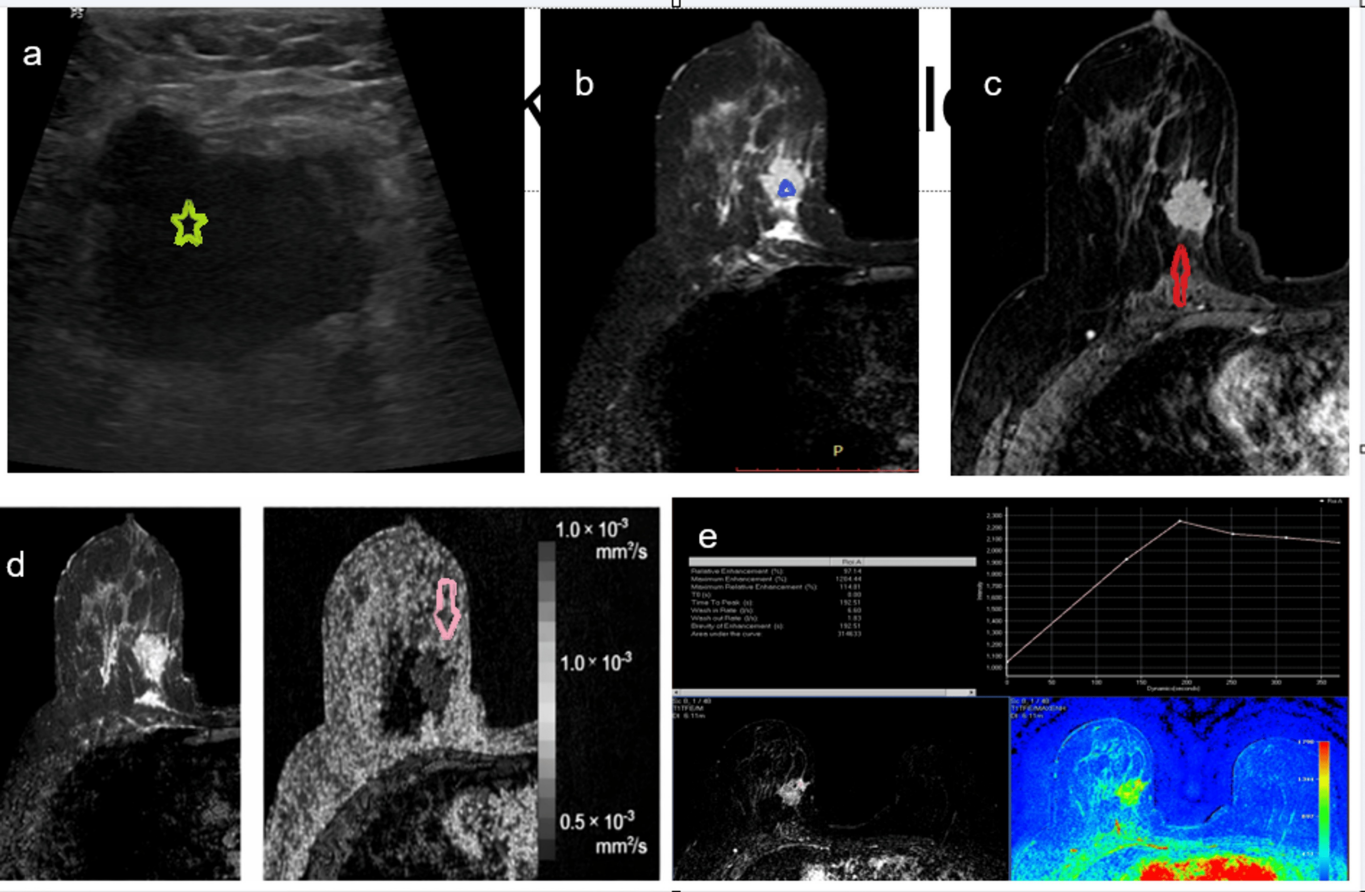
Multimodality imaging evaluation of a breast lesion (a) Ultrasound shows an irregular hypoechoic solid mass with irregular margins and no posterior acoustic features (green star). (b) T2 spectral attenuated inversion recovery (SPAIR) images demonstrate an irregular heterogeneous hyperintense mass with mild peritumoral edema (blue triangle). (c) Dynamic contrast-enhanced (DCE) MRI reveals an irregular mass with spiculate margins and heterogeneous contrast enhancement (red open arrow). (d) Diffusion-weighted imaging (DWI) and apparent diffusion coefficient (ADC) map show corresponding low ADC values within the lesion, consistent with restricted diffusion (pink open arrow). (e) DCE-MRI kinetic analysis demonstrates rapid initial enhancement with a washout pattern; the accompanying color parametric map highlights areas of maximum enhancement within the lesion.

The integration of DCE-MRI morphology, enhancement patterns, kinetic curves, and DWI significantly improves diagnostic accuracy compared with using any single modality alone [11,12]. Multiparametric breast MRI plays a pivotal role in improving the characterization of suspicious breast lesions by integrating morphological assessment with functional imaging techniques. In the present study, diffusion-weighted imaging provided valuable information regarding tissue cellularity, with lesions demonstrating diffusion restriction and corresponding low ADC values showing a higher likelihood of malignancy. Dynamic contrast-enhanced MRI further complemented these findings by evaluating lesion vascularity and enhancement kinetics, with rapid initial enhancement and plateau or washout patterns suggestive of malignant neovascularization. Figure 5 illustrates a representative case from our study demonstrating concordant findings on ultrasound, diffusion-weighted imaging, ADC mapping, and contrast enhancement kinetics, underscoring the added diagnostic value of combining DWI and DCE-MRI over morphology alone.

In this prospective study evaluating the characterization of suspicious lesions, we found that MRI outperformed ultrasonography (USG) in accurately characterizing lesions. Combining DCE-MRI with DWI allowed for high-confidence differentiation between benign and malignant lesions.

Conventional breast MRI is primarily based on combined assessment of lesion morphology and enhancement kinetics, providing high sensitivity for breast cancer detection but only moderate specificity. The ACR BI-RADS MRI lexicon standardizes evaluation of morphologic features, including shape, margin, and enhancement pattern, and kinetic characteristics, such as initial enhancement and delayed phase behaviour. While features like irregular or spiculated margins, rim or segmental enhancement, and rapid wash-in with washout are suggestive of malignancy, considerable overlap exists between benign and malignant lesions, leading to diagnostic ambiguity. To improve specificity, DWI has been incorporated into breast MRI protocols. Several studies have demonstrated the potential of DWI as an adjunct tool for differentiating benign from malignant breast lesions [13-16]. Kul et al. reported an improvement in MRI specificity to 89.2% with the combination of DCE-MRI and DWI, without a significant reduction in sensitivity (p = 1.000) [13]. Yabuuchi et al. reported a sensitivity of 92% and specificity of 86% [14], while Partridge et al. demonstrated a 10% improvement in positive predictive value with the addition of DWI to DCE-MRI [15].

Despite these advantages, standardization of diffusion-weighted breast imaging remains a challenge, particularly regarding optimal b-values and ADC thresholds. In the present study, ADC values were calculated using b-values of 0 and 1000 s/mm². Two ADC cut-off values were evaluated. At a threshold of <1.25 × 10⁻³ mm²/s, DWI achieved a sensitivity of 88%, specificity of 85%, and diagnostic accuracy of 81% (χ² = 5.7, p = 0.0006). A stricter cut-off of <1.0 × 10⁻³ mm²/s resulted in significantly improved specificity (92%) but reduced sensitivity (69%) (p = 0.016). A 2023 systematic review and meta-analysis reported a recommended mean ADC threshold of approximately 1.25 ± 0.17 × 10⁻³ mm²/s (range 0.93-1.60 × 10⁻³ mm²/s) for differentiating benign and malignant breast lesions at 1.5 T, consistent with the ADC cutoff selected in our study [17].

Multiparametric MRI approach reduces false positives, avoids unnecessary biopsies, and improves patient triage. Further large-scale and multicenter studies are recommended to validate these findings and develop standardized ADC cutoffs suited to Indian populations.

Overall, this study reinforces the role of multiparametric breast MRI in evaluating suspicious lesions and highlights the benefit of combining multiple imaging biomarkers, offering a more streamlined and objective diagnostic pathway.

This study has certain limitations, including its single-center design and relatively small sample size, which may limit the generalizability of the findings and the statistical robustness of the results. Background parenchymal enhancement was not stratified according to the phase of the menstrual cycle, which could have influenced enhancement patterns in premenopausal women. Additionally, all imaging assessments were performed by a single experienced breast radiologist; therefore, inter-observer variability was not evaluated. As the study was conducted at a single tertiary care institution, patient demographics and imaging protocols may reflect institution-specific characteristics, potentially limiting applicability to other clinical settings. Nevertheless, standardized imaging protocols were followed, and the results are concordant with existing published evidence. Larger multicenter studies with multiple readers are warranted to validate these findings.

## Conclusions

Multiparametric MRI, combining DCE-MRI morphology, enhancement patterns, kinetic curves, and DWI, provides superior accuracy over ultrasonography for characterizing suspicious breast lesions in dense breasts. While vascularity alone on color Doppler is limited, a resistive index of >0.7 remains a highly specific marker of malignancy.

These findings support the use of combined DCE-MRI and DWI as a reliable, non-invasive tool for early and accurate differentiation of benign and malignant lesions, potentially reducing unnecessary biopsies.
